# Effect of low-dose ciprofol on postoperative nausea and vomiting following gynecologic day surgery: a randomized controlled trial

**DOI:** 10.3389/fmed.2025.1612322

**Published:** 2025-09-05

**Authors:** Xinyuan Shi, Zongcai Qi, Yaxin Wei, Hongyi Xiao, Rui Zhang, Guiquan Zhuang, Meixia Zhuang, Aizhen Mou, Fangli Yue, Fanceng Ji, Peihe Nie

**Affiliations:** ^1^School of Anaesthesiology, Shandong Second Medical University, Weifang, China; ^2^Department of Anesthesiology, Weifang People’s Hospital, Weifang, China

**Keywords:** ciprofol, remimazolam, alfentanil, day surgery, postoperative nausea and vomiting

## Abstract

**Purpose:**

Low-dose propofol has a preventive effect on postoperative nausea and vomiting (PONV). Ciprofol is a new short-acting GABA_A_ receptor agonist developed in China with a similar chemical structure to propofol, but its effect on PONV is unclear. This study examines whether administration of low-dose ciprofol immediately after the start of surgery reduces the incidence of PONV.

**Patients and methods:**

In this study, this study enrolled 75 patients undergoing gynecological ambulatory surgery under general anesthesia, and randomly divided the patients into ciprofol group and normal saline control group. Both groups received remimazolam (6 mg/kg/h) until asleep, then alfentanil (20 μg/kg) and mivacurium (0.2 mg/kg) intravenous, followed by maintenance infusions of remimazolam and alfentanil. Five minutes after the start of the surgery, the ciprofol group was given 2 mL of ciprofol (5 mg) and the control group was given 2 mL of normal saline. The primary outcome measure was the incidence of PONV in the PACU. Secondary outcomes included the incidence of emetic episodes or nausea in the PACU and within 24 h, anesthetic time, wake-up time, and administered doses of remimazolam and alfentanil. Safety outcomes encompassed hypotension, hypertension, bradycardia, and tachycardia.

**Results:**

The incidence of PONV in the PACU was significantly lower in the ciprofol group compared to the control group (16.2% vs 52.6%; RR=0.31; 95% CI, 0.14–0.68; ARR=0.364; *p* = 0.002). The ciprofol group also exhibited a lower incidence of emetic episodes (defined as retching, vomiting, or both) in the PACU (RR=0.27; 95% CI, 0.10–0.75; ARR=0.287; *p* = 0.005), as well as a lower incidence of nausea (RR=0.31; 95% CI, 0.14–0.68; ARR=0.364; *p* = 0.002). The frequency of emetic episodes or nausea within 24 h postoperatively was similar between the groups. There were no significant differences between the two groups regarding anesthesia time, wake-up time, remimazolam and alfentanil dosage, or safety indicators (*p* > 0.05).

**Conclusion:**

Low-dose ciprofol can effectively prevent PONV in PACU after gynecological day surgery under general anaesthesia remazolam combined with afentanil, but its effect is limited and cannot reduce the incidence of emetic episodes or nausea within 24 h after surgery.

**Clinical trial registration:**

https://www.chictr.org.cn, identifier ChiCTR2300077247.

## 1 Introduction

Day surgery has been widely used worldwide due to its advantages of quick recovery and short hospital stay. Postoperative nausea and vomiting (PONV) is one of the most common complications after day surgery, which seriously affects patients’ postoperative recovery and quality of life (1). Remimazolam is a new type of benzodiazepine with fast onset of action and rapid recovery and can be used for the induction and maintenance of general anaesthesia (2). Alfentanil has the advantages of short action time, fast elimination, no accumulation and low incidence of PONV, and is more suitable for day surgery anaesthesia than sufentanil and remifentanil (3). However, studies have shown that the incidence of nausea and vomiting after remimazolam combined with alfentanil anesthesia is relatively high (4, 5). PONV with this type of anesthesia remains a concern.

Ciprofol is a new type of intravenous anaesthetics researched. The core structure of classical short-acting intravenous anesthetics is 2,6-disubstituted phenol, which binds to GABA_A_ receptors to produce an anesthetic effect, and propofol is the most widely used drug among these drugs (6). Ciprofol adds a cyclopropyl group to the chemical structure of propofol, which enhances its affinity with the GABA_A_ receptor and shows higher lipid solubility and potency than propofol, with less lipids from ciprofol reaching the circulatory system compared to propofol for the same level of anaesthesia (7). Additionally, ciprofol demonstrates clinical advantages including a lower incidence of injection pain and a smooth onset of action (8). Studies have shown that low-dose propofol (when not used as the primary anesthetic) has a certain anti-nausea and vomiting effect, which can effectively prevent PONV under general anaesthesia of remimazolam (9). It is still unclear whether low-dose ciprofol (when not used as the primary anesthetic) has the effect of preventing PONV.

Therefore, we conducted this randomized, controlled study,the aim of this study is to observe the effect of low-dose ciprofol (when not used as the primary anesthetic) immediately after the start of surgery reduces the incidence of PONV, so as to provide a new and effective method of preventing PONV.

## 2 Methodology

The manuscript was written in accordance with the CONSORT statement guideline for a randomized controlled trial.

### 2.1 Ethical considerations

We conducted this study in accordance with the Declaration of Helsinki. The study protocol was approved by the Ethics Committee of Weifang People’s Hospital (Ethics No. KYLL20231009-2) and registered with the China Clinical Trial Registry (ChiCTR2300077247; https://www.chictr.org.cn). From November, 2023 to January, 2024, the study was conducted in the department of anaesthesiology, Weifang People’s Hospital, Weifang City, China, where the registration was carried out and informed consent was signed with the patients.

### 2.2 Patient inclusion criteria

Patients undergoing gynecological day surgery in Weifang People’s Hospital were selected, and the inclusion criteria were ASAI-II, 18–65 years old, and BMI 18-28 kg/m^2^. Exclusion criteria were a history of preoperative sedation and analgesics, patients with allergies to the study drug, hearing abnormalities, patients with surgery time of less than 10 min or more than 1 h, patients with additional diploids after surgery, and patients with missing follow-up.

### 2.3 Randomization and blinding

Randomization was performed by an independent investigator using SPSS 25.0 software (IBM Corporation, New Orchard Road, Armonk, NY; https://www.ibm.com/products/spss-statistics) with a random number table method, allocating participants at a 1:1 ratio into either the ciprofol group or the saline control group. Patients were blinded to group assignment, while anesthesiologists were aware of their allocation. Data collection was performed by an anesthetist assistant blinded to group assignments. All anesthetic procedures were administered by the same anesthesia team.

### 2.4 Methods of anaesthesia

All patients complied with standardized preoperative fasting protocols: a minimum fasting duration of 6–8 h for solid foods and ≥ 2 h for clear fluids before anesthesia induction. Forearm venous access is established after the patient is admitted, and a multifunctional monitor (mindray BeneVision N17) was connected to monitor the electrocardiogram (ECG), noninvasive blood pressure (NIBP), heart rate (HR), pulse saturation (SpO_2_), and anaesthesia depth index (BIS). Dexamethasone 5 mg and flurbiprofen 50 mg were administered before induction of anaesthesia.

Both groups were induced by remimazolam 6 mg/kg/h infusion until falling asleep (call without opening eyes), followed by alfentanil 20ug/kg and mivacurium chloride 0.2 mg/kg injected slowly (30 s each). After 3 min of assisted respiration, a laryngeal mask was placed and mechanical ventilation maintained ETCO_2_ 35–45 mmHg. Anaesthesia was maintained by continuous intravenous remimazolam 1 mg/kg/h and alfentanil 40ug/kg/h to maintain BIS values between 40 and 60. In the ciprofol group, 2 mL of ciprofol (5 mg) was given at 5 min after the start of surgery. In the control group, 2 mL of saline was given at 5 min after the start of surgery.

Bradycardia (HR < 50 beats min^−1^ for at least 1 min) and hypotension (SBP below <90 mmHg or ≥30% lower than preoperative or mean arterial pressure <60 mmHg) are treated symptomatically with atropine 0.3 mg or ephedrine 6 mg during surgery. Esmolol was given to treat tachycardia (HR > 100 beats min^−1^ for at least 1 min) and hypertension (SBP above >140 mmHg or ≥30% higher than preoperative or mean arterial pressure >110 mmHg) was decreased with urapidil. The drug was stopped at the end of the surgery, and When the patient wakes up, the laryngeal mask was removed, and the patient was sent to the post-anaesthesia care unit (PACU). Patients can be returned to the ward after being observed in the PACU for at least 30 min and having an Aldrete score of ≥ 9. Discharged after meeting the discharge criteria of PADS≥9.

All patients were followed up by a blinded evaluator on the second day after surgery through a questionnaire or a telephone form,and they need to answer two questions: Q1. Have you vomited or had dry-retching? Q2. Have you experienced a feeling of nausea (“an unsettled feeling in the stomachand slight urge to vomit”)? If yes, has your feeling of nausea interfered with activities of daily living, such as being able to get out of bed, being able to move about freely in bed, being able to walk normally, or eating and drinking (10)?

Basic information about the patients was obtained through preoperative anaesthesia clinic assessment, intraoperative anaesthesia monitoring, inpatient electronic medical records, and observation records in the PACU.

### 2.5 Observation indicators

The primary outcome was the incidence of PONV in the PACU. Secondary outcomes included the incidence of emetic episodes or nausea in the PACU and within 24 h, anesthetic time, wake-up time, and administered doses of remimazolam and alfentanil. Safety outcomes included hypotension (SBP below <90 mmHg or ≥30% lower than preoperative or mean arterial pressure <60 mmHg), hypertension (SBP above >140 mmHg or ≥30% higher than preoperative or mean arterial pressure >110 mmHg), bradycardia (HR < 50 beats min^−1^ for at least 1 min), tachycardia (HR > 100 beats min^−1^ for at least 1 min) during anaesthesia.

### 2.6 Sample size calculation

PASS 15.0 software (https://www.ncss.com/online-store/) was used for sample size calculation, assuming a typeIerror of 0.05 (bilateral) and a test efficacy of 90%. Based on preliminary trial results, 3 of 22 patients (13.6%) in the ciprofol group experienced nausea and vomiting, whereas 10 of 21 patients (47.6%) in the normal saline group developed these symptoms, and the sample size was calculated to be at least 34 cases in each group, taking into account a possible 15% loss to follow-up, resulting in the final inclusion of 80 patients, 40 cases in each group.

### 2.7 Statistical analysis

In this study, SPSS 25.0 software (IBM Corporation, New Orchard Road, Armonk, NY; https://www.ibm.com/products/spss-statistics) was used for statistical analysis of data. Data distribution was assessed using the ShapiroeWilk test. Continuous variables with a normal distribution are expressed as means (SD), non-normal variables are reported as medians [interquartile range (IQR)] and count data results are expressed as frequency (%). Comparisons between groups were performed using the chi-square test, Fisher’s exact test, or rank-sum test. The prophylaxis effect of ciprofol vs. control was assessed by Risk Ratio (RR) and its 95% confidence interval (CI). A two-sided *p* < 0.05 was considered as the difference was statistically significant.

## 3 Results

### 3.1 Patient condition

**Figure 1:**
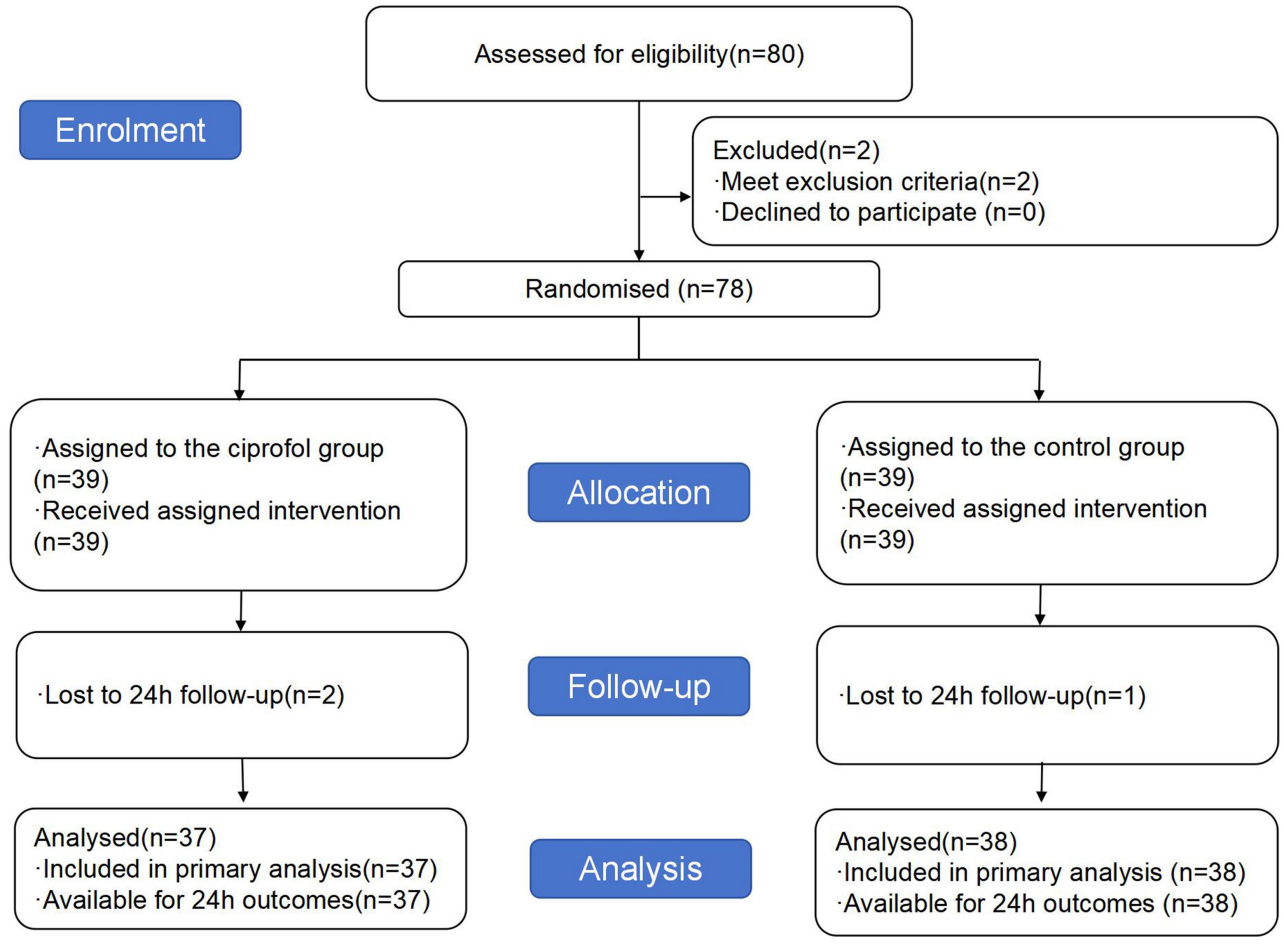
Flow chart of the experiment.

The recruitment period was from November 2023 to January 2024, a total of 80 patients were recruited (Figure 1), 2 patients were excluded (One patient was excluded due to analgesic use for lower abdominal pain, while another was excluded for regularly taking sedative medication before sleep to manage chronic insomnia), 78 patients were randomly included in the study, due to 3 patients lost follow-up, and finally 75 patients were included in the statistical analysis.

### 3.2 Baseline and perioperative characteristics

The baseline characteristics of the two subject groups were balanced in Table 1. The median age (IQR) was 40 (35.00–49.00) years in the ciprofol group and 41(35.25–48.00) years in the control group. Body weight and BMI were comparable between groups, with the ciprofol group showing a mean body weight of 59.97 kg and a median BMI (IQR) of 22.95 (22.03–24.14) kg/m^2^, while the control group had a mean body weight of 58.51 kg and a median BMI (IQR) of 22.24 (20.15–24.85) kg/m^2^. All subjects were classified as ASA class 1–2. Preoperative Apel scores were evenly distributed, with 21 patients scoring 1–2 points and 16 scoring 3–4 points in the ciprofol group, compared to 23 and 15 patients in the control group, respectively. Additionally, no significant difference was observed in surgical categories between the two groups.

**Table 1:** General information of patients.

Variables	Ciprofol group (*n* = 37)	Control group (*n* = 38)	*P*
Age [years, M (Q1 ~ Q3)]	40 (35.00–49.00)	41 (35.25 to 48.00)	0.981
Weight (kg, x ± S)	59.97 (4.18)	58.51 (6.84)	0.272
BMI [kg/m^2^, M(Q1 ~ Q3)]	22.95 (22.03 to 24.14)	22.24 (20.15 to 24.85)	0.350
ASA [example (%)]			
I	7 (18.9)	8 (21.1)	0.819
II	30 (81.1)	30 (78.9)	
Apfel score [example (%)]			
1–2	21 (56.8)	23 (60.5)	0.742
3–4	16 (43.2)	15 (39.5)	
Surgery category [example (%)]			
Hysteroscopy	29 (78.4)	28 (73.7)	0.636
Conization	8 (21.6)	10 (26.3)	

Data are presented as mean (SD), median (interquartile range), or *n* (%).

BMI, body mass index; ASA, American society of anesthesiologists; PONV, postoperative nausea and vomiting.

### 3.3 Primary outcome

The incidence of PONV in the PACU in the ciprofol group was 16.2% significantly lower than 52.6% in the control group [6 (16.2%) vs 20 (52.6%); RR = 0.31; 95% CI, 0.14–0.68; ARR = 0.364; *p* = 0.002] (Table 2).

**Table 2:** Primary, secondary, and safety outcomes.

	Ciprofol group (*n* = 37)	Control group (*n* = 38)	Risk ratio (95% CI)	*P*
Primary outcome				
PONV in the PACU [example (%)]	6 (16.2)	20 (52.6)	0.31 (0.14 to 0.68)	0.002*
Secondary outcomes				
Emetic episodes [example (%)]				
PACU	4(10.8)	15 (39.5)	0.27 (0.10 to 0.75)	0.005*
0-24 h	8(21.6)	15 (39.5)	0.55 (0.26 to 1.14)	0.084
Nausea [example (%)]				
PACU	6 (16.2)	20 (52.6)	0.31 (0.14 to 0.68)	0.002*
0-24 h	19 (51.4)	21(55.3)	0.93 (0.61 to 1.42)	0.736
Anaesthesia time [min, M (Q1 ~ Q3)]	20.00 (14.00 to 28.00)	23.00 (18.25 to 29.00)	-	0.146
Wake-up time [min, M (Q1 ~ Q3)]	6.00 (5.00 to 7.00)	6.00 (5.00 to 6.75)	-	0.382
Remimazolam dosage [mg, M (Q1 ~ Q3)]	18.00 (11.00 to 27.80)	16.50 (12.13 to 22.83)	-	0.463
Alfentanil dosage [mg, M (Q1 ~ Q3)]	0.72 (0.44 to 1.04)	0.56 (0.42 to 0.88)	-	0.214
Safety outcomes				
Hypotension [example (%)]	8(21.6)	7 (18.4)	1.17 (0.47 to 2.91)	0.781
Hypertension [example (%)]	2(5.4)	0	-	0.149
Bradycardia [example (%)]	2(5.4)	5 (13.2)	0.41 (0.09 to 1.99)	0.430
Tachycardia [example (%)]	1(2.7)	1 (2.6)	1.03 (0.07 to 15.82)	1.000

Data are median (interquartile range) or *n* (%). *Significance difference in comparison with control group (*P* < 0.05).

PONV, postoperative nausea and vomiting; CI, confidence interval.

### 3.4 Secondary outcomes

**Figure 2:**
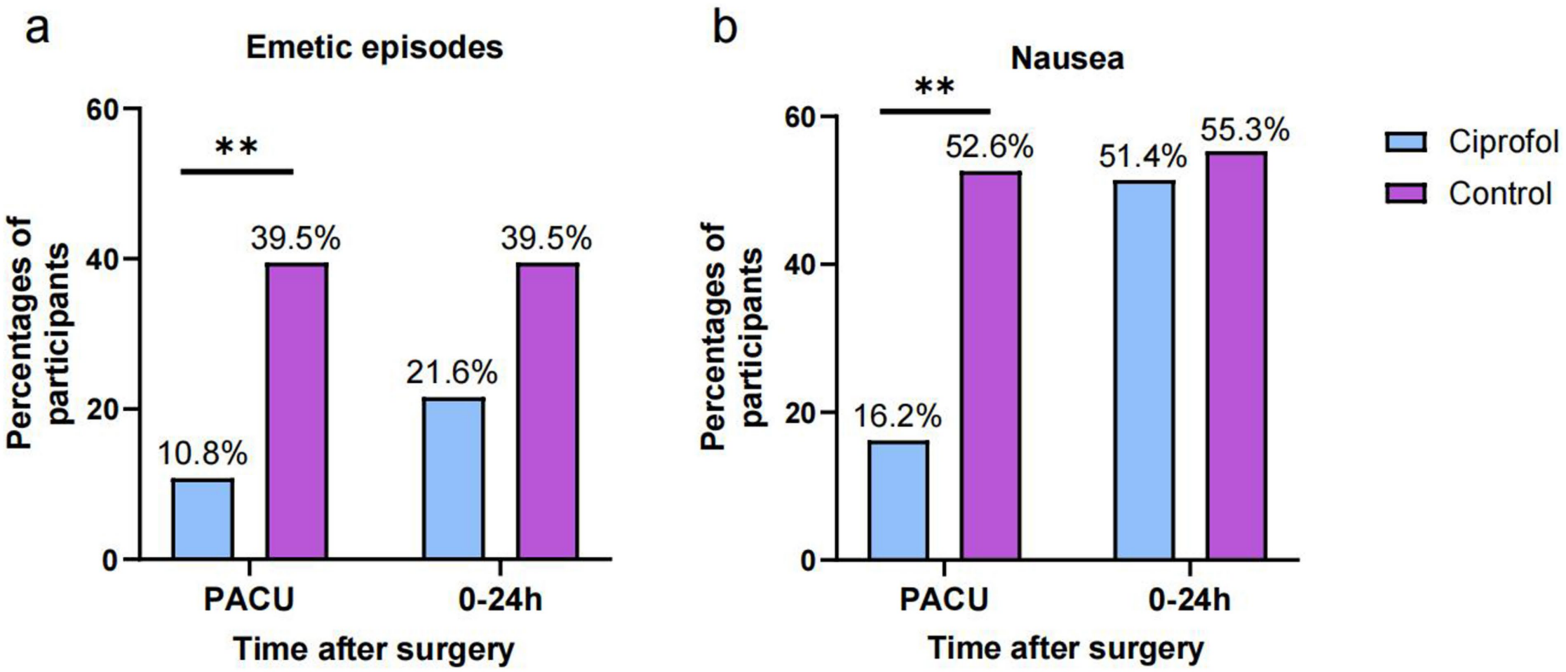
The rates of emetic episodes and nausea in the PACU and during 0–24 after surgery. **(A)** the rates of emetic episodes; **(B)** the rates of nausea; Significance was determined by two-tailed X2 test. *p < 0.05; **p < 0.01.

Outcomes for nausea or emetic episodes are shown in Table 2 and Figure 2. The occurrence frequency of emetic episodes in the ciprofol group was lower in the PACU [4 (10.8%) vs 15 (39.5%); RR = 0.27; 95% CI, 0.10–0.75, ARR = 0.287; *p* = 0.005], The occurrence frequency of nausea in the ciprofol group was lower in the PACU [6 (16.2%) vs 20 (52.6%); RR = 0.31; 95% CI, 0.14–0.68; ARR = 0.364; *p* = 0.002]. The frequency of nausea or emetic episodes was similar in the 24 h after surgery. There was no significant difference in anesthesia time, wake-up time, remimazolam and alfentanil dosage between the two groups (*p* > 0.05).

### 3.5 Safety outcomes

There was no significant difference in safety outcomes between the two groups (*p* > 0.05). Hypotension occurred in 8 cases (21.6%) in the ciprofol group and 7 cases (18.4%) in the control group. Hypertension and tachycardia are uncommon (Table 2).

## 4 Discussion

In adult patients undergoing gynecological ambulatory surgery, we report for the first time that adding low-dose ciprofol to remimazolam-alfentanil anesthesia significantly reduces both the incidence and severity of PONV in the PACU. However, no significant efficacy was observed for emetic episodes or nausea within 24 h postoperatively. These findings suggest that low-dose ciprofol provides short-term prophylaxis against PONV, offering clinical benefits in high-volume ambulatory centers with constrained PACU resources through accelerated turnover, optimized nursing allocation, and reduced hospitalization costs. The lack of sustained antiemetic effect beyond 24 h may be attributable to its pharmacokinetic profile. Sole reliance on low-dose ciprofol for PONV prophylaxis could compromise patient comfort, necessitating combination therapy with longer-acting antiemetics for extended protection.

PONV is one of the common complications after anaesthesia and surgery, and women are one of the important risk factors for the occurrence of PONV (11). Most patients undergoing hysteroscopy surgical treatment are young women, and the occurrence of PONV after hysteroscopy surgery may be related to the large release of neurotransmitters such as 5-hydroxytryptamine caused by postoperative uterine contraction pain and the use of intraoperative opioids, which can stimulate the body’s chemoreceptor trigger zone and vomiting centre, causing the nausea and vomiting reflex (12). Day surgery has a short duration, but adverse reactions such as PONV can affect patients’ postoperative recovery, and how to reduce the incidence of PONV is important for improving patient prognosis and patient recovery.

Remimazolam belongs to the new short-acting benzodiazepine sedatives, which are metabolized by non-specific plasma esterase hydrolysis, with a rapid onset of action, short half-life, and rapid awakening after discontinuation (13). However, Yuji Suzuki and colleagues in a single-centre retrospective cohort study found a higher incidence of PONV after remimazolam anaesthesia than propofol anaesthesia (5). Alfentanil is a fentanyl derivative acting at the *μ* receptor, a short-acting strong opioid analgesic with the advantages of fast onset, short duration of action, rapid elimination, and no accumulation, and it is suitable for use in gynaecological hysteroscopy surgery in which the operative time is short and the rhythm is fast, and it has a low incidence of PONV when compared with sufentanil and fentanyl (3).

Propofol is one of the most commonly used intravenous anaesthetics and can directly inhibit the vagal nucleus, the central emetic chemoreceptor area, and can also act as an antiemetic through the modulation of subcortical structures and pathways (14). Borgeat and colleagues concluded that the antiemetic effect of propofol can be exerted in concentrations lower than the sedative effect (15). A study of the Cechetto DF found that the reduced levels of serotonin in the area postrema and the cerebrospinal fluid may explain the antiemetic property of propofol, propofol may also directly act on area postrema neurons via a GABA_A_ receptor to reduce their activity (16). Ciprofol is a new short-acting GABA_A_ receptor agonist. The introduction of a cyclopropyl group to the chemical structure of propofol results in a chiral structure that increases the steric effect and thus enhances its affinity for the GABA_A_ receptor (6). Ciprofol is approximately 4–6 times more potent than propofol, with a smooth onset of action, and a very low incidence of injection pain (8). Our team’s studies have confirmed that ciprofol has a similar anaesthetic effect in gynaecological day surgery compared to propofol, and that the incidence of adverse events was lower in the ciprofol group (17). This study revealed a 16.2% incidence of PONV in the PACU with low-dose ciprofol, aligning with our team’s prior findings demonstrating that low-dose propofol significantly reduces PACU PONV incidence (10.6%) following remimazolam-alfentanil anesthesia (9). Furthermore, an outpatient hysteroscopy study showed comparable PONV incidence between ciprofol and propofol groups at both 1 h and 24 h postoperatively (18). Collectively, these observations suggest ciprofol may share similar mechanisms with propofol for PONV prophylaxis, warranting further pharmacokinetic investigations to elucidate the underlying pharmacological basis.

In this study, we found that there was no significant difference in the incidence of emetic episodes or nausea within 24 h after surgery, and it can be inferred that ciprofol has a limited time to prevent emetic episodes or nausea and cannot effectively reduce the occurrence and severity of emetic episodes or nausea within 24 h. This is similar to the results of the study of Xiao HY in the study of low-dose propofol for the prevention of nausea or vomiting after general anaesthesia with remimazolam and alfentanil (9). This phenomenon may be attributed to ciprofol’s pharmacokinetics, characterized by triexponential elimination with corresponding half-lives of 2.0 min (t_1/2_,*α*), 34.9 min (t_1/2_,*β*), and 6.2 h (t_1/2_,*γ*). The clinical effects demonstrate dose dependency (19, 20). Potentially explaining why low-dose ciprofol provides only transient antiemetic efficacy. Clinically, proactive co-administration with antiemetics is warranted to prevent prolonged emetic episodes or nausea. Furthermore. The occurrence of emetic episodes or nausea may increase in patients due to the possibility of using transportation such as cars when they are discharged from the hospital within 24 h. This study also suggests that continued attention should be given to the prevention of emetic episodes or nausea in patients undergoing gynaecological day surgery. Further studies are needed to determine whether the mechanism of action of ciprofol in preventing emetic episodes or nausea is similar to that of propofol. There was no significantly difference in the wake-up time between the two groups, indicating that low-dose ciprofol given during surgery did not prolong the wake-up time.

This study still has some limitations. Firstly, this study was a single-centre study with a relatively small sample size, which may have some selection bias, and the influencing factors of PONV were not further analysis, and it is necessary to further validate the present results in a multi-centre, large-sample study. Secondly, this study had a relatively short observation period, and a longer follow-up observation period is needed, and patient satisfaction or recovery quality score surveys need to be increased to gain a more comprehensive understanding of patient recovery. Thirdly, we only recruited gynecological day surgery patients, which limits the universality,and it is necessary to expand the range of surgical types for further research. Fourth, while this study studies the short-term effects of a single dose of low-dose ciprofol administration, future systematic pharmacokinetic studies are warranted to comprehensively characterize its metabolic trajectory and elimination profile.

## 5 Conclusion

In conclusion,low-dose ciprofol can effectively prevent PONV in PACU after gynecological day surgery under general anaesthesia remazolam combined with afentanil, but its effect is limited and cannot reduce the incidence of emetic episodes or nausea within 24 h after surgery. This finding underscores the importance of implementing a full-period antiemetic strategy during day surgery. Future research should focus on clarifying its mechanism, exploring the synergistic effects with long-acting antiemetic drugs, and verifying their application value in a wider population.

## Data Availability

The original contributions presented in the study are included in the article/supplementary material, further inquiries can be directed to the corresponding author/s.

## References

[1] Gran Bruun AM Svensen K Johansen E Halstensen TD Gustavsson A Leonardsen ACL. A quantitative, multicentre, longitudinal study of patient experiences after gynaecological day surgery. Nurs Open. (2023) 10:1536–44. doi: 10.1002/nop2.1403, PMID: 36210540 PMC9912434

[2] Schüttler J Eisenried A Lerch M Fechner J Jeleazcov C Ihmsen H. Pharmacokinetics and pharmacodynamics of remimazolam (CNS 7056) after continuous infusion in healthy male volunteers: part I. Pharmacokinetics and clinical pharmacodynamics. Anesthesiology. (2020) 132:636–51. doi: 10.1097/ALN.0000000000003103, PMID: 31972655

[3] Li Z Xiao H Zhu T Yue F Fu M Ji T. Observation on the application effect of alfentanil compounded with propofol in anaesthesia for daytime hysteroscopy surgery. J Shandong Med. (2022) 62:72–5. doi: 10.3969/j.issn.1002-266X.2022.09.018

[4] Yi F Xiao H Zhu T Man Y Ji F. Prevention of postoperative nausea and vomiting after gynaecological day surgery under remimazolam general anesthesia: a randomized double-blind controlled study. BMC Anesthesiol. (2022) 22:292 2022 Sep 15. doi: 10.1186/s12871-022-01835-x, PMID: 36109691 PMC9476338

[5] Suzuki Y Kawashima S Makino H Doi M Nakajima Y. Comparison of postoperative nausea and vomiting between remimazolam and propofol: a propensity score-matched, retrospective, observational, single-center cohort study. Korean J Anesthesiol. (2023) 76:143–51. doi: 10.4097/kja.22441, PMID: 36245344 PMC10079003

[6] Lu M Liu J Wu X Zhang Z. Ciprofol: A Novel Alternative to Propofol in Clinical Intravenous Anesthesia? Biomed Res Int. (2023) 2023:7443226. doi: 10.1155/2023/7443226 36714027 PMC9879693

[7] Liao J Li M Huang C Yu Y Chen Y Gan J. Pharmacodynamics and pharmacokinetics of HSK3486, a novel 2,6-disubstituted phenol derivative as a general Anesthetic. Front Pharmacol. (2022) 13:830791. doi: 10.3389/fphar.2022.830791, PMID: 35185584 PMC8851058

[8] Chen BZ Yin XY Jiang LH Liu JH Shi YY Yuan BY. The efficacy and safety of ciprofol use for the induction of general anesthesia in patients undergoing gynecological surgery: a prospective randomized controlled study. BMC Anesthesiol. (2022) 22:245. doi: 10.1186/s12871-022-01782-7, PMID: 35922771 PMC9347095

[9] Xiao H Liu M Man Y Wei Y Ji F. Effect of low-dose propofol combined with dexamethasone on the prevention of postoperative nausea and vomiting in gynaecological day surgery under remimazolam-based general anesthesia. Medicine (Baltimore). (2023) 102:e33249. doi: 10.1097/MD.0000000000033249, PMID: 36897701 PMC9997807

[10] Myles PS Wengritzky R. Simplified postoperative nausea and vomiting impact scale for audit and post-discharge review. Br J Anaesth. (2012) 108:423–9. doi: 10.1093/bja/aer505, PMID: 22290456

[11] Choy R Pereira K Silva SG Thomas N Tola DH. Use of Apfel simplified risk score to guide postoperative nausea and vomiting prophylaxis in adult patients undergoing same-day surgery. J Perianesth Nurs. (2022) 37:445–51. doi: 10.1016/j.jopan.2021.10.006, PMID: 35305914

[12] Shaikh SI Nagarekha D Hegade G Marutheesh M. Postoperative nausea and vomiting: a simple yet complex problem. Anesth Essays Res. (2016) 10:388–96. doi: 10.4103/0259-1162.179310, PMID: 27746521 PMC5062207

[13] Hu Q Liu X Wen C Li D Lei X. Remimazolam: an updated review of a new sedative and anaesthetic. Drug Des Devel Ther. (2022) 16:3957–74. doi: 10.2147/DDDT.S384155, PMID: 36411859 PMC9675580

[14] Kampo S Afful AP Mohammed S Ntim M Buunaaim ADB Anabah TW. Sub-hypnotic dose of propofol as antiemetic prophylaxis attenuates intrathecal morphine-induced postoperative nausea and vomiting, and pruritus in parturient undergoing cesarean section - a randomized control trial. BMC Anesthesiol. (2019) 19:177. doi: 10.1186/s12871-019-0847-y, PMID: 31521119 PMC6745062

[15] Borgeat A Wilder-Smith OH Saiah M Rifat K. Subhypnotic doses of propofol possess direct antiemetic properties. Anesth Analg. (1992) 74:539–41. doi: 10.1213/00000539-199204000-00013, PMID: 1554120

[16] Cechetto DF Diab T Gibson CJ Gelb AW. The effects of propofol in the area postrema of rats. Anesth Analg. (2001) 92:934–42. doi: 10.1097/00000539-200104000-00027, PMID: 11273930

[17] Man Y Xiao H Zhu T Ji F. Study on the effectiveness and safety of ciprofol in anesthesia in gynecological day surgery: a randomized double-blind controlled study. BMC Anesthesiol. (2023) 23:92. doi: 10.1186/s12871-023-02051-x, PMID: 36964501 PMC10039513

[18] Zhang H Zhang M Hao L Li Q Li Q Yang J. Comparison of the effects of Ciprofol and propofol on postoperative nausea and vomiting in patients undergoing outpatient hysteroscopy. Drug Des Devel Ther. (2024) 18:5701–7. doi: 10.2147/DDDT.S489223, PMID: 39659951 PMC11628403

[19] Bian Y Zhang H Ma S Jiao Y Yan P Liu X. Mass balance, pharmacokinetics and pharmacodynamics of intravenous HSK3486, a novel anaesthetic, administered to healthy subjects. Br J Clin Pharmacol. (2021) 87:93–105. doi: 10.1111/bcp.14363, PMID: 32415708

[20] Expert Panel. Clinical practice guidelines for ciprofol administration. Chin J Anesthesiol. (2021) 41:129–32. doi: 10.3760/cma.j.cn131073.20201011.00201

